# In Vitro Model for Studying Esophageal Epithelial Differentiation and Allergic Inflammatory Responses Identifies Keratin Involvement in Eosinophilic Esophagitis

**DOI:** 10.1371/journal.pone.0127755

**Published:** 2015-06-03

**Authors:** Kiran KC, Marc E. Rothenberg, Joseph D. Sherrill

**Affiliations:** Department of Pediatrics, University of Cincinnati College of Medicine, Division of Allergy and Immunology, Cincinnati Children’s Medical Center, Cincinnati, Ohio, United States of America; University of Central Florida, UNITED STATES

## Abstract

Epithelial differentiation is an essential physiological process that imparts mechanical strength and barrier function to squamous epithelia. Perturbation of this process can give rise to numerous human diseases, such as atopic dermatitis, in which antigenic stimuli can penetrate the weakened epithelial barrier to initiate the allergic inflammatory cascade. We recently described a simplified air-liquid interface (ALI) culture system that facilitates the study of differentiated squamous epithelia in vitro. Herein, we use RNA sequencing to define the genome-wide transcriptional changes that occur within the ALI system during epithelial differentiation and in response to allergic inflammation. We identified 2,191 and 781 genes that were significantly altered upon epithelial differentiation or dysregulated in the presence of interleukin 13 (IL-13), respectively. Notably, 286 genes that were modified by IL-13 in the ALI system overlapped with the gene signature present within the inflamed esophageal tissue from patients with eosinophilic esophagitis (EoE), an allergic inflammatory disorder of the esophagus that is characterized by elevated IL-13 levels, altered epithelial differentiation, and pro-inflammatory gene expression. Pathway analysis of these overlapping genes indicated enrichment in keratin genes; for example, the gene encoding keratin 78, an uncharacterized type II keratin, was upregulated during epithelial differentiation (45-fold) yet downregulated in response to IL-13 and in inflamed esophageal tissue from patients. Thus, our findings delineate an in vitro experimental system that models epithelial differentiation that is dynamically regulated by IL-13. Using this system and analyses of patient tissues, we identify an altered expression profile of novel keratin differentiation markers in response to IL-13 and disease activity, substantiating the potential of this combined approach to identify relevant molecular processes that contribute to human allergic inflammatory disease.

## Introduction

The stratified squamous epithelium provides a protective barrier against environmental insult to the underlying mucosa. This essential function is mediated, in part, through the well-programmed process of epithelial differentiation, whereby proliferating basal cells migrate through the suprabasal layers and enter a terminally differentiated senescent state once at the luminal surface [1]. The basal and suprabasal layers of the epithelium can be uniquely characterized by the expression of different types of epithelial keratins (KRT), which form a network of intermediate filaments that add structural strength to the epithelium [2]. Keratin intermediate filaments are formed by the equimolar polymerization of acidic type I and basic type II keratins, which are composed of an N-terminal head domain, a C-terminal tail domain, and an alpha helical rod domain that is responsible for dimerization [2]. Keratins exhibit specific expression patterns within the stratified squamous epithelium. For instance, the type I keratin cytokeratin 5 (KRT5) and its type II interacting partner cytokeratin 14 (KRT14) are expressed in undifferentiated basal epithelial cells, whereas the KRT4/13 pair is expressed in the differentiated epithelial cells of the suprabasal layers [3].

Altered keratin distribution and/or function have been associated with multiple atopic epithelial barrier disorders such as atopic dermatitis (AD). Notably, *KRT5*, whose expression is restricted to the basal layer in normal epidermis, is expressed in the suprabasal layer of the epidermis in AD lesions [4]. Moreover, mutations in *KRT5* and/or *KRT14* are known to cause epidermolysis bullosa simplex (EBS), which is marked by skin blisters and cell fragility of basal keratinocytes [5]. Eosinophilic esophagitis (EoE) is a chronic, allergic inflammatory disorder of the esophagus that is characterized by interleukin 13 (IL-13)–mediated esophageal epithelial cell differentiation and barrier defects [6–10]. We have shown that IL-13 specifically downregulates desmoglein 1 (*DSG1*), a desmosomal cadherin that indirectly binds to the intracellular keratin network, and that *DSG1* downregulation is sufficient to drive epithelial barrier dysfunction [6, 11]. In a recent pre-clinical trial of adult patients with EoE, anti-IL-13 therapy was demonstrated to be effective in reducing esophageal eosinophil levels and normalizing disease-associated transcript signatures, including increased *DSG1* and decreased *KRT14* and *KRT16* levels [10]; however, the direct regulation of esophageal epithelial cell keratins by IL-13 in the context of EoE remains unaddressed.

In the present study, we describe the development of a simplified air-liquid interface (ALI) culture system and a global molecular characterization of the key markers of differentiated and stratified esophageal epithelium. We demonstrate that, under homeostatic conditions, the ALI culture system recapitulates a strikingly similar gene expression profile to that of healthy esophageal tissue in vivo. Moreover, we show that the presence of IL-13 in the ALI culture system induces an overlapping gene signature and the disease-associated pathways observed in the inflamed esophageal mucosa of patients with EoE. The expression of epithelial keratins, in particular the uncharacterized type II keratin *KRT78*, was negatively regulated by IL-13 and in EoE patient tissues. Our findings demonstrate an esophageal epithelial cell keratin gene network involving *KRT78* that is dysregulated by IL-13 and in EoE patient tissues and highlight the ALI culture system as a useful in vitro tool to study esophageal epithelial developmental processes and allergic inflammatory responses.

## Materials and Methods

### Cell lines

The immortalized human esophageal epithelial cell line (EPC2-hTERT) (kindly provided by Anil Rustgi, MD, University of Pennsylvania) were cultured as previously described [6, 12].

### Patient demographics

All patients analyzed in this study provided written consents and all studies were approved by the Institutional Review Board at Cincinnati Children’s Hospital Medical Center (CCHMC) (#2008–0090). Patients analyzed by RNA sequencing have been characterized previously [7]. In brief, patients with active EoE were defined as having a previous EoE diagnosis while being unresponsive to proton pump inhibitor therapy and having greater than 15 eosinophils per high-powered microscopic field (HPF) within a distal esophageal biopsy. Patients with EoE in disease remission were defined as having a previous EoE diagnosis and having less than 15 eosinophils/HPF within a distal esophageal biopsy while undergoing dietary therapy (elimination or elemental) or on swallowed glucocorticoids at the time of biopsy. Healthy (NL) controls had no previous diagnosis of EoE and no eosinophils within a distal esophageal biopsy.

### Air-liquid interface (ALI) culture system

The ALI differentiation protocol was performed as previously described [6]. Briefly, immortalized esophageal epithelial cells (EPC2-hTERT) were seeded onto semi-permeable membranes (0.4 μm) and grown to confluence in the presence of low-calcium media (keratinocyte serum-free media, 0.09 mM calcium). Epithelial differentiation was induced by the addition of extracellular calcium (1.8 mM final concentration) over the course of five days (day 3 to 8). Stratification was induced by removing the media from the upper chamber and exposing the cells to the ALI for a period of 6 days (day 8 to 14). Treatment with IL-13 (100 ng/mL) in the lower chamber occurred at the start of the ALI exposure (day 8), with fresh media plus IL-13 added every 3 days until day 14. Cells were then collected for RNA and histology.

### RNA sequencing analyses

RNA sequencing analysis was performed by the CCHMC Genetic Variation and Gene Discovery Core. In brief, RNA was isolated using the RNeasy kit (QIAGEN Incorporated, Germantown, MD) according to the manufacturer’s protocol. Whole-transcriptome (RNA) sequencing was performed at the CCHMC Gene Discovery and Genetic Variation Core as previously described [7]. Sequencing reads were aligned against the GRCh37 genome model using TopHat 2.04 with Bowtie 2.03 [13, 14]. The separate alignments were then merged using Cuffmerge [15] with UCSC gene models as a reference. Raw data were assessed for statistical significance using a Welch t-test (esophageal biopsies) or ANOVA with Tukey post-hoc test (ALI samples) with Benjamini-Hochberg false discovery rate and a threshold of *P* < 0.05 and a 2.0-fold cut-off filter in GeneSpring GX (Agilent Technologies Incorporated, Santa Clara, CA). RNA sequencing data were deposited into the Gene Expression Omnibus (GEO) (GSE58640 and GSE65335).

### Quantitative PCR

RNA isolated from the ALI samples were used for cDNA synthesis (iScript, BioRad, Hercules, CA). Quantitative PCR analysis using SYBR Green was performed (BioRad, Hercules, CA). Primers used for amplification were as follows: *KRT78*, forward (TGGACTTCAGCAGCATCATC) and reverse (CTGGGCAGACACCTGAAGTT); *IL13*, forward (ACAGCCCTCAGGGAGCTCAT) and reverse (TCAGGTTGATGCTCCATACCAT). All data were normalized to the housekeeping gene *GAPDH* as previously described [16]. Normalized data are presented as mean with SEM and were assessed for statistical significance using an ANOVA with Tukey’s multiple comparison tests and unpaired t-test with a significance threshold of *P* < 0.05.

### Cloning and transfection

*KRT78* was cloned in frame with the GFP into pEGFPN1 vector at *Eco*RI and *Bam*HI site using the primers (GCGAATTCGGACCATGTCTCTCTCCCCATGCCG) and (GCGGATCCCGGTAGGTGATGGATGT), respectively. Transfections were performed using *Trans*IT-LT1 transfection reagent (Mirus Bio, Madison, WI).

### Protein sequence alignment and analysis

Primary protein sequence alignment was retrieved from the Human Intermediate Filament Database (www.interfil.org). Cladogram construction and phylogenetic analysis were performed using http://phylogeny.lirmm.fr/ as described [17].

### Immunofluorescence and imaging

Transduced EPC2-hTERT cells were grown on chamber slides (Thermo Scientific, Waltham, MA), and immunofluorescent staining for DSG1 and nuclear staining with DAPI was performed as previously described [6] and imaged using an Olympus B51x system.

### Western blotting

Protein lysates were prepared from biopsy tissues using M-PER mammalian protein extraction reagent (Thermo Scientific, Rockford, IL) with protease inhibitors and by sonication and centrifugation. BCA assay (Thermo Scientific, Rockford, IL) was used to quantify protein. Lane Marker Reducing Sample Buffer (5X; Thermo Scientific, Rockford, IL) was added to the soluble protein fraction after centrifugation, and 6 μg of protein was loaded in SDS page gels (Invitrogen, Carlsbad, CA). Protein was transferred to a nitrocellulose membrane, blocked in Odyssey blocking buffer (LI-COR biosciences, Lincoln, NB), and probed with rabbit anti-KRT78 antibody (PA5-26855; Thermo Fisher Scientific, Rockford, IL) and rabbit anti-GAPDH (#2118; Cell Signaling Technology, Danvers, MA) at 1:1000 each in Odyssey blocking buffer. Following primary antibody incubation, membranes were probed with goat anti-Rabbit IRDye 800CW (926–3211; LI-COR biosciences, Lincoln, NB) and imaged using a LiCOR Odyssey CLX system.

## Results

### Gene expression in the ALI reflects that of human esophageal tissue

**Fig 1:**
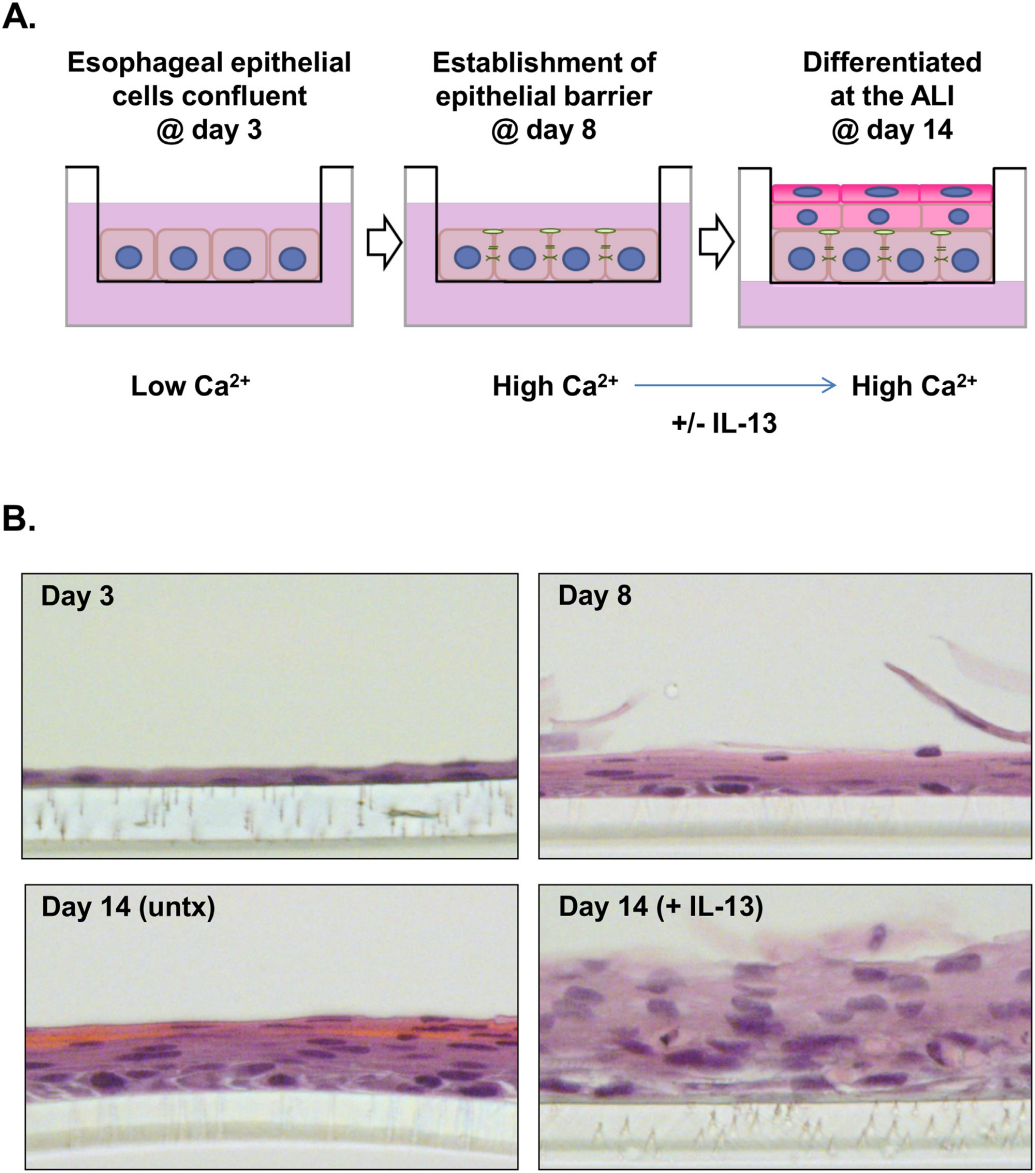
The ALI culture system. Schematic diagram depicting the air-liquid interface (ALI) culturing protocol (A). Representative images (40X) of hematoxylin- and eosin-stained sections at various time points and/or treatments during the ALI protocol (B). The clear semi-permeable membrane can be seen in the bottom of all images. Untx, untreated.

The immortalized human esophageal epithelial cell line EPC2-hTERT [12] was subjected to ALI culture as depicted schematically in Fig 1A. Histologic analysis at various time points over the course of the ALI culture demonstrated the development of an eosin-stained layer of multilayered cells with flattened morphology (e.g., differentiated epithelial cells) at day 14 compared to day 8, suggesting the development of stratified differentiated epithelium (Fig 1B). In the presence of prolonged IL-13 exposure, striking morphological changes occurred as reported previously [6], including reduced epithelial differentiation and expansion of the epithelial layer (day 14 [+ IL-13] vs. day 14 [untreated]) (Fig 1B).

**Fig 2:**
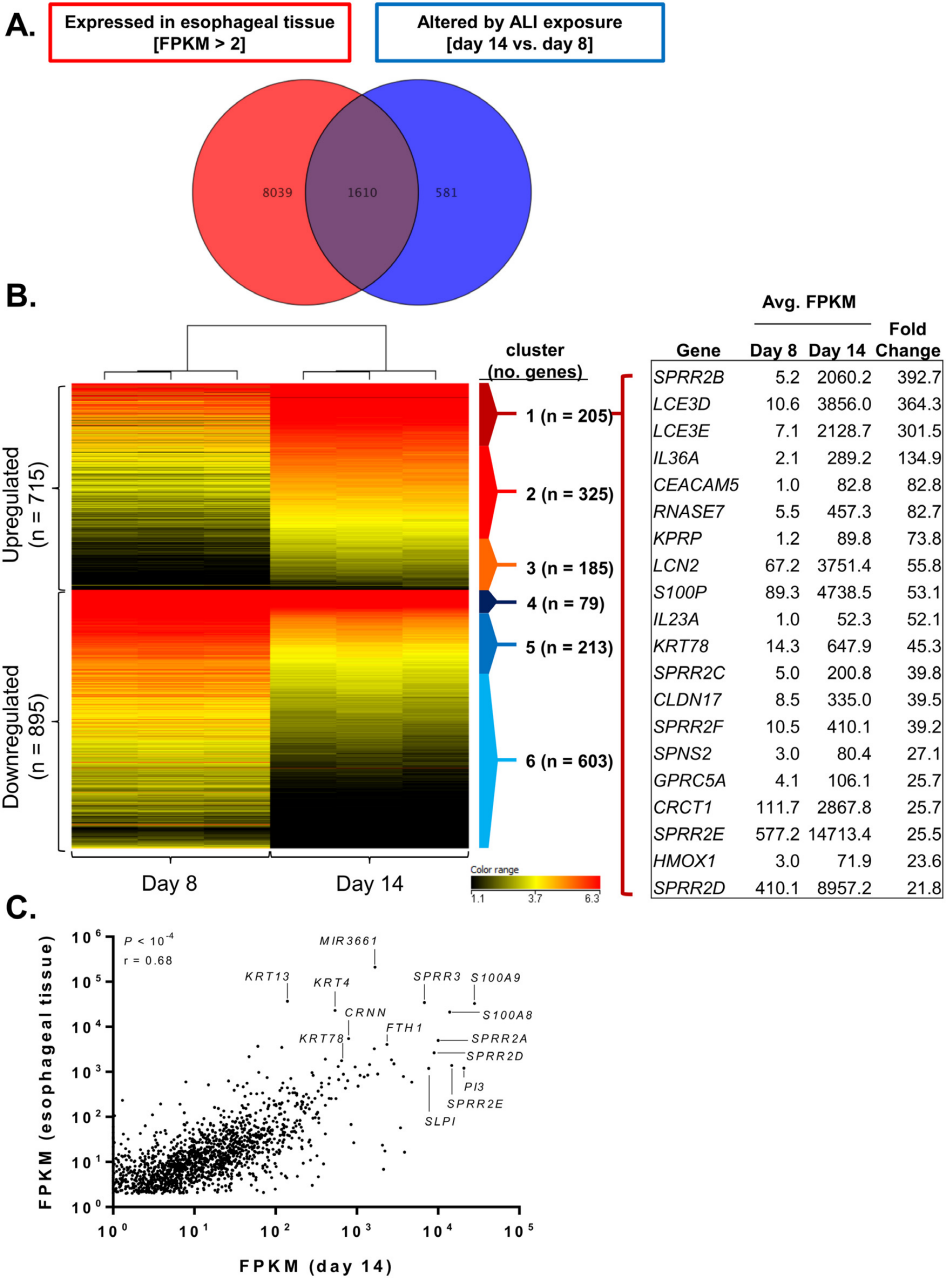
In vitro ALI differentiation replicates gene signature of healthy tissue ex vivo. Venn diagram depicting the number of genes expressed in healthy esophageal tissue (FPKM > 2, n = 9,649) and the number of genes altered during air-liquid interface (ALI) differentiation (day 14 compared to day 8, n = 2,191) (*P* < 0.05, fold change > 2.0) (A). Clustered heatmap showing the log_2_ FPKM values of the 1,610 genes in common to both data sets. Also shown are the 20 most dysregulated genes in cluster 1 (induced at day 14) (B). Expression (FPKM) correlation for the 1,610 genes in common between the healthy esophageal tissue and genes altered during ALI differentiation (C).

In order to assess the potential for the ALI culture system in replicating differentiated esophageal epithelium at the molecular level, RNA sequencing was performed on esophageal epithelial cells at day 8 and day 14 of the ALI system. A total of 2,191 genes were significantly dysregulated upon ALI differentiation (*P* < 0.05, fold change > 2.0). When compared to the RNA sequencing profile of the 9,649 genes expressed in healthy esophageal tissue (FPKM > 2), over 73% (1,610 out of 2,191) of the genes dysregulated upon ALI differentiation were found to also be expressed in healthy esophageal tissue (Fig 2A). The 1,610 genes formed six distinct clusters: those that were induced at high (cluster 1; n = 205), medium (cluster 2; n = 325), or low (cluster 3; n = 185) levels at day 14 compared to day 8 and those that were repressed at high (cluster 4; n = 79), medium (cluster 5; n = 213), or low (cluster 6; n = 603) levels at day 14 compared to day 8 (Fig 2B). Importantly, the most highly induced genes at day 14 (in cluster 1) (e.g., *SPRR and LCE* gene family members) are primarily restricted to differentiated epithelial cells, supporting the ALI model as an in vitro model for differentiated epithelium (Fig 2B). Remarkably, the expression levels of the 1,610 genes induced by ALI exposure and overlapping with healthy esophageal tissue expression correlated with the relative expression pattern (i.e., upregulated or downregulated) in healthy esophageal tissue (*P* < 10^–4^, Spearman r = 0.68), with many of the most highly expressed genes in both data sets belonging to gene families located in the epidermal differentiation cluster (EDC) on chromosome 1q21 [18] (e.g., *S100A8* and *S100A9* and *SPRR2A*, *SPRR2D*, and *SPRR2E*) (Fig 2C).

### Keratin gene signature in differentiated esophageal epithelial cells

**Fig 3:**
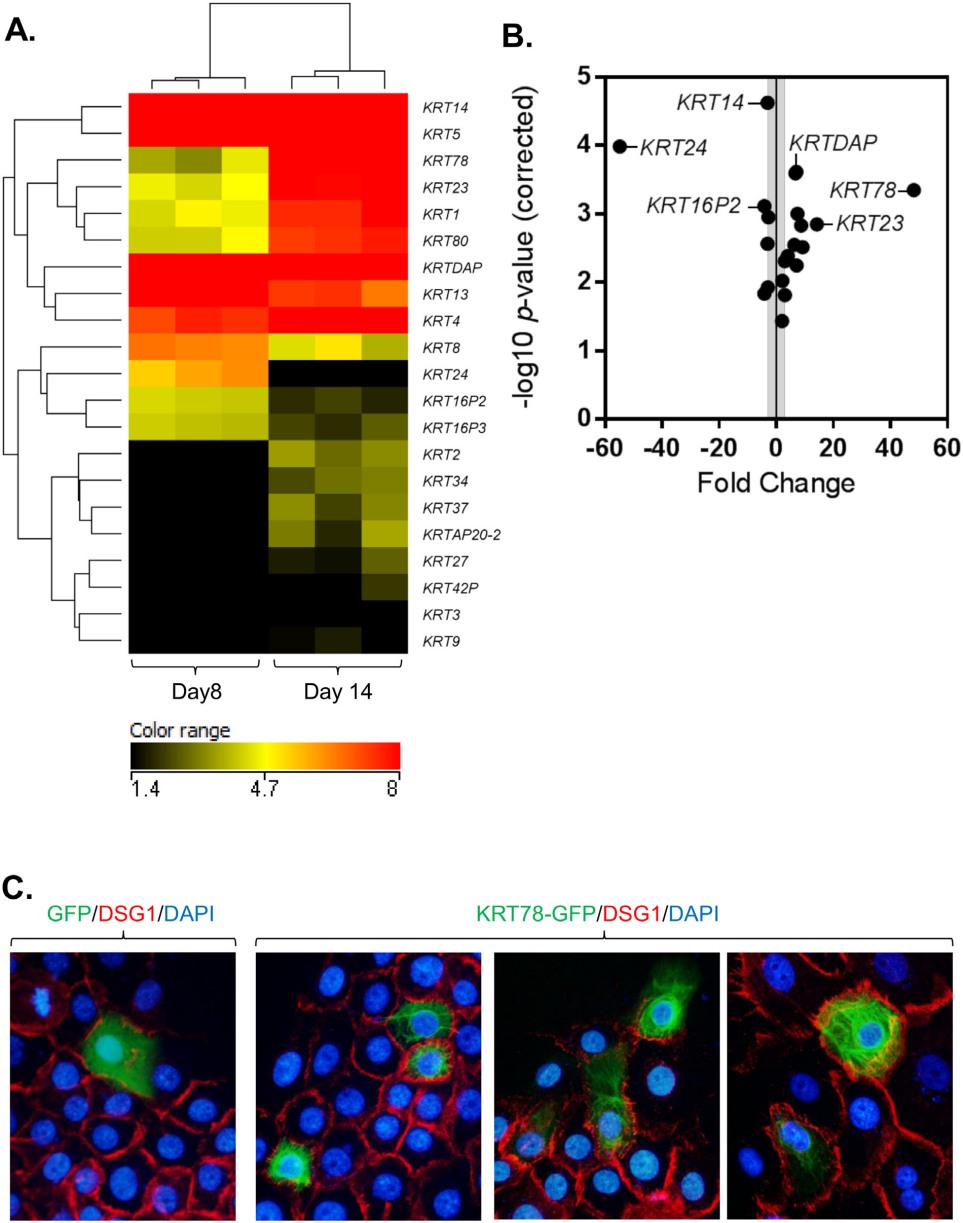
Differential keratin expression during differentiation with ALI culture. Clustered heatmap showing the log_2_ FPKM values (A) and volcano plot showing the fold change and—log_10_ *P*-values (B) of the 21 keratin gene family members significantly changed in the air-liquid interface (ALI) culture system at day 14 compared to day 8 (*P* < 0.05, fold change > 2). Immunofluorescence staining of EPC2-hTERT cells transfected with GFP (empty vector control) or KRT78-GFP (green) (C). Cells were co-stained for DSG1 (red) and nuclei are stained with DAPI (blue). Images are representative of 3 independent experiments and taken at 400X magnification.

Of the 1,610 genes altered upon ALI differentiation and also expressed in healthy esophageal tissue, a group of keratin genes were of particular note. In all, RNA sequencing identified 21 keratin genes that had significantly altered expression at day 14 compared to day 8 (*P* < 0.05, fold change >2.0) (Fig 3A). Of those 21 genes, 14 had increased expression at day 14 compared to day 8. Keratin 78 (*KRT78*), an uncharacterized type II epithelial keratin, was the most upregulated (48 fold), and *KRT24*, a type I epithelial keratin, was the most downregulated (55 fold) with ALI differentiation (Fig 3B).

**Fig 4:**
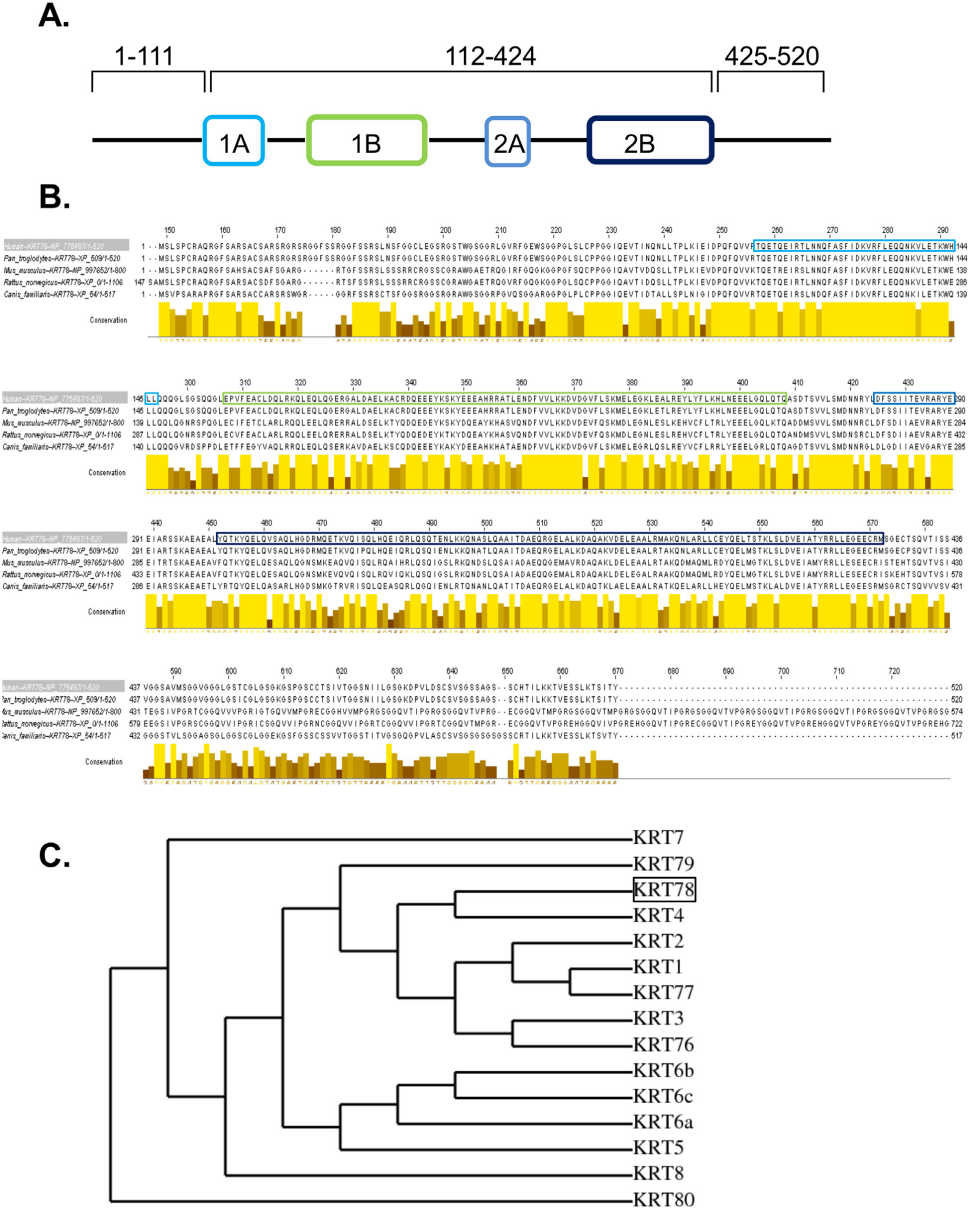
Domain and conservation analysis of keratin 78. Protein sequence and domain structure of KRT78, a type II keratin (A). Primary protein sequence alignment of KRT78 across species showing conservation in the coiled-coil alpha helical domains and the flanking non-helical head and tail domains (B). Cladogram showing the evolutionary relationship among all type II epithelial keratins (constructed using maximum likelihood) (C).

Despite its abundant expression and dynamic regulation in differentiating epithelium, little insight into *KRT78* (originally classified as keratin 5b) has been reported since its initial discovery in 2005 [19, 20]. We first sought to define the localization of KRT78 in esophageal epithelial cells. Overexpression of GFP-tagged keratin 78 in EPC2-hTERT cells showed a filamentous network of KRT78 distribution throughout the cytoplasm (Fig 3C) reflecting that of other type II keratins [21]. Indeed, using the Human Intermediate Filament Mutation Database (http://www.interfil.org), domain structure analysis showed that KRT78 has a canonical keratin structure consisting of a head region (amino acids 1–111), coiled-coil region (amino acids 112–424), and tail region (amino acids 425–520) (Fig 4A). Primary sequence alignment of KRT78 protein demonstrated a striking conservation among higher mammals, particularly within the coiled-coiled region (Fig 4B). In an effort to gain insight into the potential function of KRT78, we performed phylogenetic analysis of all type II epithelial keratins and identified KRT78 as most closely related to keratin 4 (KRT4), which is downregulated in esophageal squamous cell carcinoma [22] (Fig 4C).

### Allergic inflammation alters keratin expression in vitro and in vivo

**Fig 5:**
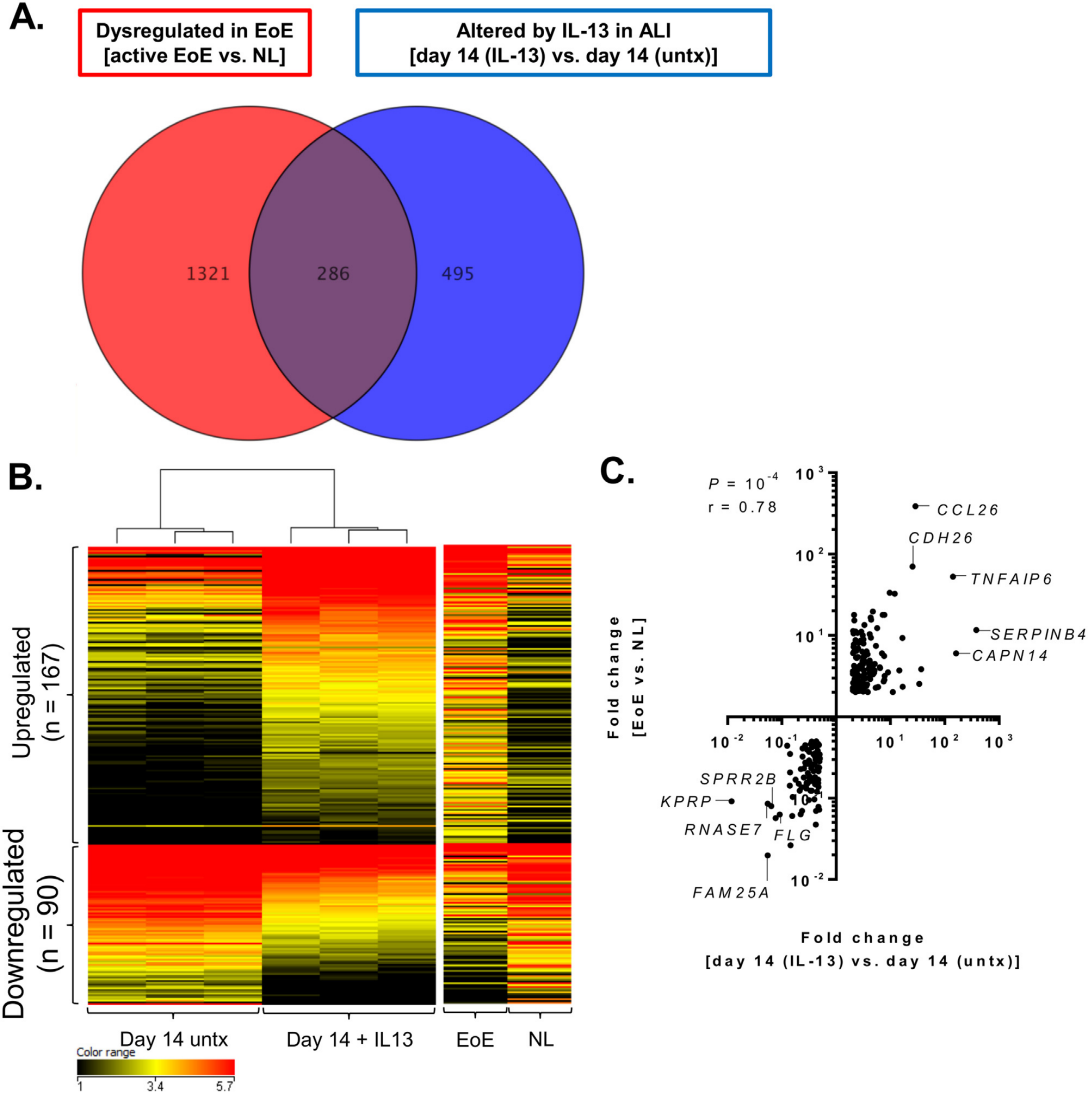
Identification of disease-associated genes in the ALI system. Venn diagram depicting the number of genes dysregulated in the inflamed esophageal tissue of patients with active eosinophilic esophagitis (EoE) compared to healthy (NL) controls (*P* < 0.05, fold change > 2, n = 1,607) and the number of genes altered in the air-liquid interface (ALI) after prolonged IL-13 exposure (day 14 [IL-13] compared to day 14 [untx]; *P* < 0.05, fold change > 2; n = 781) (A). Clustered heatmap showing the log_2_ FPKM values (B) and fold change correlation (C) of the 257 genes similarly dysregulated in the ALI after prolonged IL-13 exposure and in the inflamed esophageal tissue of patients with active eosinophilic esophagitis (EoE). Untx, untreated.

We next investigated the ability of the ALI culture system to model chronic allergic inflammation ex vivo. RNA sequencing on ALI-differentiated cells at day 14 treated with IL-13 (100 ng/mL, day 8 through 14) identified 781 genes that were significantly altered by IL-13 (*P* < 0.05, fold change > 2.0) compared to untreated cells at day 14 (Fig 5A). When compared to the RNA sequencing profile of genes dysregulated in inflamed esophageal tissue from patients with active EoE compared to healthy controls (*P* < 0.05, fold change > 2.0; n = 1,607), 37% (286 out of 781) of the genes altered by IL-13 in the ALI were identified (Fig 5A). Of these 286 genes, 257 genes were similarly upregulated or downregulated in both EoE and by IL-13 in the ALI culture system and formed 5 distinct clusters (Fig 5B). Notably, the regulation of many of these genes were opposite of that observed during ALI differentiation (day 14 vs. day 8), such that genes that were highly induced in the ALI at day 14 were inhibited during prolonged IL-13 exposure and in EoE (Fig 5B); the 10 most dysregulated genes within each cluster are listed with fold changes in Table 1. The fold changes of all 257 genes were also significantly correlated between EoE and ALI-differentiated cells treated with IL-13 (*P* < 10^–4^, Spearman r = 0.78) (Fig 5C). Many of the most highly correlated genes, such as *CCL26*, *TNFAIP6*, *CDH26*, and *CAPN14* have been previously identified as IL-13–regulated genes [23–25]. Conversely, the downregulated genes were composed largely of epithelial differentiation genes. For instance, *KPRP* was highly induced at ALI day 14 (71 fold compared to day 8) yet almost completely inhibited by IL-13 in the ALI and reduced in EoE by 85 fold and 11 fold, respectively (Table 1).

**Table 1:** Fold change of top 10 genes induced and top 10 genes inhibited in the ALI after prolonged IL-13 exposure and altered in EoE.

	Gene symbol	Fold change [day 14 untx]^1^	Fold change [day 14 + IL-13]^2^	Fold change [EoE]^3^
**Induced**	*SERPINB4*	-5.2	379.3	11.6
	*CAPN14*	-3.1	161.7	6.0
	*TNFAIP6*	N/A	141.0	52.7
	*FETUB*	N/A	37.0	3.9
	*SERPINB3*	-4.4	33.9	2.5
	*CCL26*	N/A	28.9	387.1
	*CDH26*	2.4	25.4	70.0
	*IFIT1*	N/A	16.8	2.3
	*LBH*	N/A	16.6	9.3
	*NTRK1*	N/A	14.5	3.7
**Inhibited**	*KPRP*	70.5	-84.8	-10.9
	*FAM25A*	3.6	-18.3	-50.6
	*RNASE7*	82.8	-18.2	-11.7
	*SPRR2B*	389.7	-15.6	-12.5
	*IGFL1*	-15.3	-13.0	-17.6
	*FLG*	10.9	-10.9	-15.9
	*AX747517*	4.8	-8.0	-2.3
	*RNF223*	9.2	-7.1	-7.0
	*MT2A*	-3.5	-7.1	-4.8
	*IVL*	N/A	-7.1	-2.9

^1^Fold change at day 14 untreated as compared to day 8 (see Fig 2)

^2^Fold change at day 14 + IL-13 as compared to day 14 untreated (see Fig 3)

^3^Fold change in active EoE as compare to healthy (NL) controls (see Fig 3)

**Fig 6:**
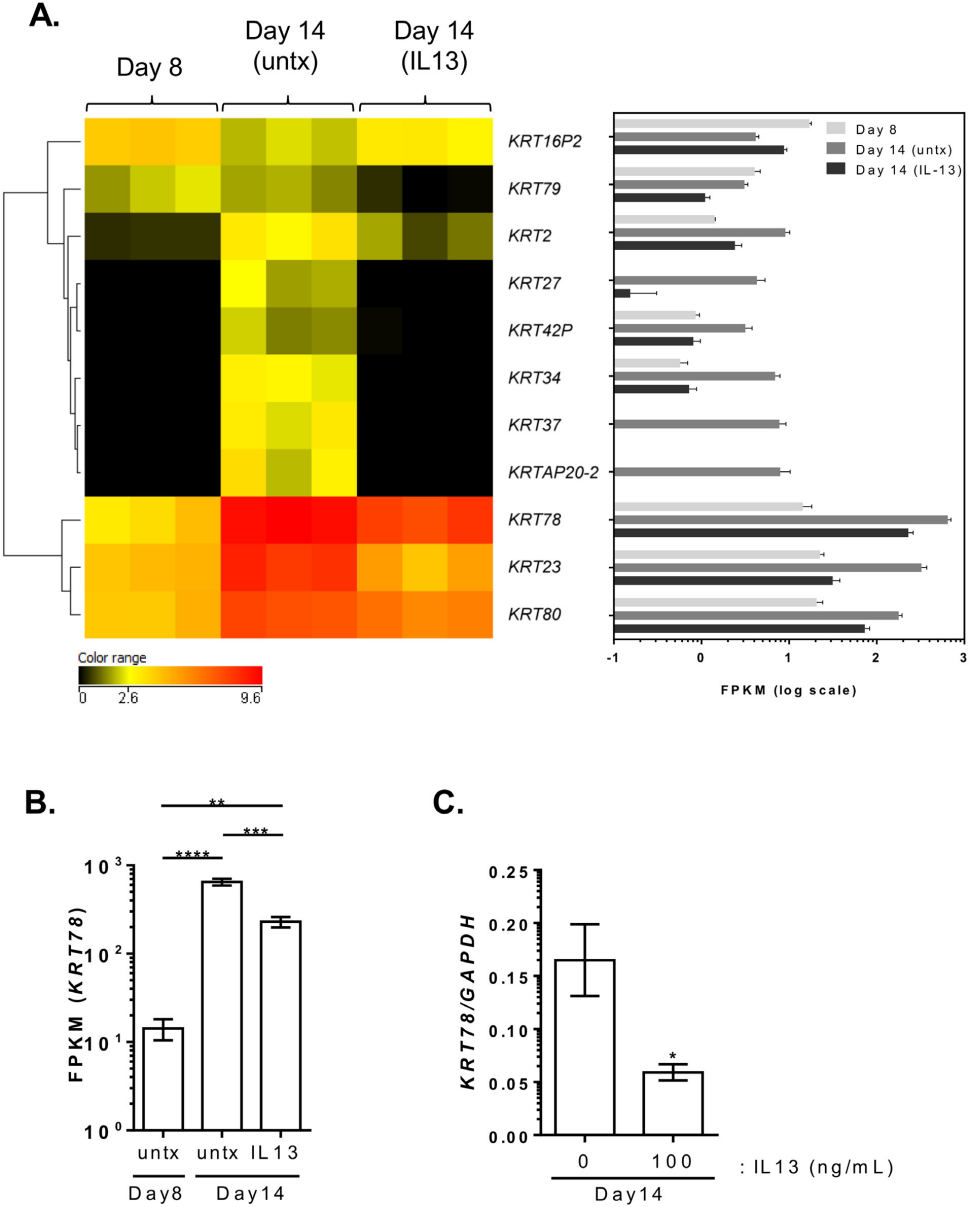
Altered keratin expression with IL-13 in the ALI differentiated tissue. Heatmap showing the log2 FPKM values of 11 keratin genes that are significantly changed with IL-13 exposure compared to untreated at day 14 (*P* < 0.05, fold change > 2) (A). FPKM values of *KRT78* from RNA sequencing data at day 8 and at day 14 treated with or without IL-13 (B). Quantitative PCR analysis of *KRT78* expression levels in differentiated ALI (day 14) tissue in the absence (0 ng/mL) or presence (100 ng/mL) of IL-13 (C). Data in (B) and (C) are represented as mean with standard error of mean (SEM). *, *P* < 0.05; **, *P* < 5 × 10–3; ***, *P* < 10–3; ****, *P* < 10–4. Untx, untreated.

**Fig 7:**
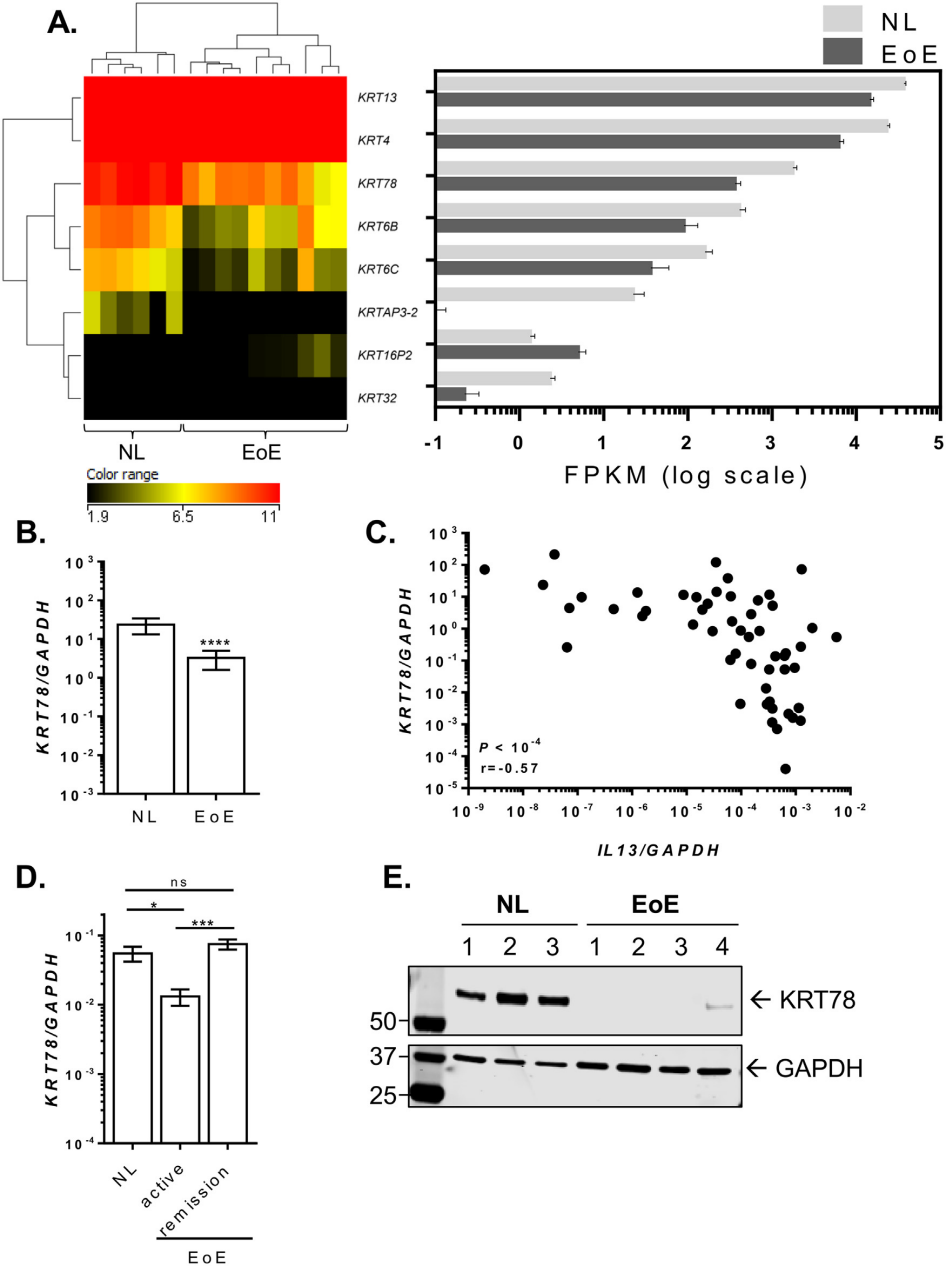
Decreased KRT78 expression in EoE. Clustered heatmap showing the log_2_ FPKM values of differentially expressed keratins in the esophagus of healthy controls (NL) and patient with active EoE (*P* < 0.05, fold change > 2.0) (A). The right panel depicts the FPKM of corresponding keratin genes in log scale. Quantitative PCR analysis of *KRT78* expression in esophageal biopsies from NL (n = 23) and patients with active EoE (n = 49) (B). Correlation between esophageal expression levels of *KRT78* and *IL13* in NL (n = 23) and patients with active EoE (n = 34) (C). Quantitative PCR analysis of *KRT78* expression in esophageal biopsies from NL (n = 11) and patients with active EoE (n = 12) and in disease remission (n = 11) (D). Western blot analysis of KRT78 (upper panel) and GAPDH (lower panel) expression in esophageal biopsy protein lysates from NL (n = 3) and patients with active EoE (n = 4) (E). Data in (B) and (D) are represented as mean with SEM. *, *P* < 0.05; ***, *P* < 10^–3^; ****, *P* < 10^-4^. Ns, not significant.

To better understand the role of keratins in the inflamed microenvironment *in vitro*, we further explored the expression of keratins in the presence of IL-13 and in inflamed esophageal biopsies from patients with EoE. Ten of the 21 keratins that were induced with ALI were inhibited by prolonged IL-13 exposure (*P* < 0.05, fold change >2) (Fig 6A). In particular, epithelial keratins such as KRT78, which was downregulated 2.8 fold, as well as *KRT80*, *KRT23*, *KRT78*, *KRT2*, and *KRT79* were downregulated by IL-13. Quantitative PCR analysis showed that the induction of *KRT78* during ALI differentiation was significantly attenuated with IL-13 treatment, consistent with FPKM reads from RNA sequencing (Fig 6C). RNA sequencing data for altered keratin gene expression in patients with active EoE was compared to the control individuals. Epithelial keratins *KRT6B*, *KRT6C*, and *KRT78* were downregulated 8.3, 5.6, and 6.1 fold, respectively, in EoE, whereas the keratin pseudogene *KRT16P2* was the only keratin-related gene that was upregulated (2.8 fold) in disease (Fig 7A). Further expression analysis by quantitative PCR on a larger cohort of patients with EoE and controls confirmed a significant reduction in *KRT78* expression in EoE (Fig 7B). Notably, *KRT78* expression showed a significant negative correlation with *IL13* expression levels (*P* < 10^–4^, Spearman r = 0.57) (Fig 7C). Interestingly, in patients with EoE in disease remission after steroid treatment or diet therapy, esophageal expression of *KRT78* normalized to the levels observed in healthy controls (Fig 7D). Western blot on esophageal biopsy lysates demonstrated lower KRT78 protein expression in patients with active EoE compared to controls (Fig 7E).

## Discussion

We have used RNA sequencing to characterize the global transcriptional changes that occur during esophageal epithelial differentiation and allergic inflammation using an in vitro ALI culture system [6, 25]. Our findings support the ALI culture system provides a robust model for studying the molecular and phenotypic changes that occur during epithelial differentiation and chronic inflammatory responses in the esophagus. Specifically, we observed a remarkable overlap (73%) in gene expression between the ALI-differentiated esophageal epithelium in vitro and healthy esophageal tissue ex vivo. Moreover, our data demonstrate that prolonged IL-13 stimulation in vitro regulates differentiated epithelial gene expression that partially mimics that of human allergic inflammation, as 37% of the IL-13 regulated transcriptome overlapped with the disease associated transcriptome in-vivo. Our ALI expression data identified a previously unrecognized dysregulation of the esophageal epithelial keratin network that included the dynamic regulation of *KRT78*, an uncharacterized type II keratin which increases during esophageal epithelial differentiation and is negatively regulated by IL-13. This particular gene was dynamically regulated in human allergic inflammatory tissue as a function of disease activity, consistent with its expression pattern in vitro.

Several in vitro models involving ALI exposure have been demonstrated to induce differentiation of epidermal, pulmonary, and esophageal epithelial cells. For instance, 3D organotypic models utilizing ALI exposure of epithelial cells grown on collagen plugs infused with fibroblasts have been a gold standard for studying the mechanisms regulating epithelial differentiation [26]. Though collagen-based models are particularly useful for assessing intercellular crosstalk during disease processes, such as epithelial to mesenchyme transition, which occurs in EoE, these models are labor intensive and utilize numerous reagents [27–29]. Collagen-based models also lack a means by which to assess differentiation throughout the course of the experiment, such as the formation of an intact epithelial barrier as measured by transepithelial resistance, which is prohibitive due to the high resistance of the underlying collagen plug; investigators are thus reliant upon histological analysis of the epithelium after the experiment. Thus, the ALI culture system can have certain advantages over collagen-based organotypic models. First, the ALI culture system yields a stratified, differentiated epithelium in approximately 2 weeks with minimal maintenance and reagents. Moreover, by growing cells directly on a semi-permeable membrane, the ability to measure epithelial barrier function is preserved [6] and, in our experience, there is less inter- and intra-experimental variation compared to collagen-based methods.

Advanced culture systems such as 3D organotypic and ALI cultures have provided in vitro evidence for mechanisms responsible for altered epithelial differentiation during several human diseases, including those of the esophagus [30]. For instance, overexpression of the transcription factors cdx1 and c-myc in esophageal epithelial cells grown in 3D organotypic culture demonstrated their capacity to induce transdifferentiation towards a Barrett’s esophagus phenotype [31]. Moreover, histamine was shown to downregulate the expression of the epidermal differentiation genes encoding filaggrin and loricrin, as well as induce stratum corneum thinning and barrier dysfunction in skin organotypic cultures [32]. Our gene expression data from the ALI culture system after prolonged IL-13 exposure demonstrating a 37% overlap with the gene signature of the inflamed esophageal tissue of patients with EoE, which largely comprises differentiated epithelial cell-specific genes, substantiates the effectiveness of our ALI culture system in replicating some of the molecular pathology associated with EoE. Although the percentage of the EoE transcriptome covered by the ALI data (286/1,607 genes = 18%) was slightly less compared to previous microarray analyses comparing IL-13–treated primary esophageal epithelial cells in standard submerged culture with the EoE-associated gene signature (126/574 genes = 22%) [23], several differences between the two studies are noteworthy. First, recent RNA sequencing from patient biopsies has nearly tripled the previous EoE transcriptome from 574 to 1,607 dysregulated genes [7]. Second, the current and prior in vitro studies used different sources of esophageal epithelial cells (immortalized EPC2-hTERT vs. primary cells, respectively). Importantly, when also taking into account the magnitude of dysregulation of genes associated with ALI differentiation (day 14 compared to day 8), the effect of a single stimulus (IL-13) on genes expressed in differentiated epithelium is quite striking. For instance *KPRP*, which is located within the EDC on 1q21 and encodes for keratinocyte proline-rich protein [33], was inhibited to almost pre-ALI differentiation (e.g., day 8) levels (Table 1). Interestingly, though little is known regarding its function, *KPRP* is highly expressed in differentiated epidermal keratinocytes and its expression is increased in psoriatic skin lesions [33].

Gene expression data from the ALI and inflamed esophageal tissue has underscored *KRT78* as a prominent esophageal epithelial keratin gene that is negatively regulated during allergic inflammation. Here we have shown that *KRT78*, a type II keratin predominantly present in the stratified epithelia of the esophagus is expressed at lower levels in EoE and is regulated by IL-13 in vitro. IL-13, as well as IL-4, has recently been shown to downregulate *DSG1*, *KRT1*, and *KRT10*, which impaired structural integrity in human keratinocytes [34]. *KRT4*, the keratin most evolutionarily similar to KRT78 in humans, is highly expressed in the esophagus and downregulated in esophageal squamous cell carcinoma [35]. Interestingly, mice deficient in keratin 4 (*Krt4* ^*-/-*^) exhibit basal cell hyperplasia in the esophagus, a key histopathological change associated with EoE, as well as a disrupted esophageal barrier that was susceptible to bacterial invasion [36, 37]. Interestingly, retinoic acid stimulation of oral epithelial cells reduced both KRT4 and DSG1 expression [38]. We have previously shown that the esophageal epithelial barrier is perturbed in EoE and in ALI-differentiated cells following IL-13 treatment, likely owing to the loss of *DSG1* [6]. Thus, it is conceivable that the negative regulation of both *KRT78* and *DSG1* synergistically drive IL-13-induced epithelial differentiation barrier defects in EoE.

In summary, these data have provided critical insight into the genetic pathways that are regulated during epithelial differentiation and inflammatory responses. In using inflamed esophageal tissue from patients with EoE as a model disease, we have shown the ALI system has recapitulated esophageal inflammatory pathways observed in vivo. When coupled with future in vivo experiments, the ALI culture system will provide an invaluable in vitro tool for analyzing critical genes, such as *KRT78*, in the pathophysiology of EoE.
